# The NHS 10-year plan: rethinking the delivery of primary care medical education

**DOI:** 10.3399/BJGP.2025.0349

**Published:** 2025-09-01

**Authors:** Jaspal S Taggar, Julie Carson, Rakesh Patel

**Affiliations:** 1 Primary Care Education Unit, University of Nottingham, Nottingham, UK; 2 Institute of Health Sciences Education, Queen Mary University of London, London, UK

The recent investigation by Lord Darzi into the state of the English NHS highlighted the need for service redesign while also recognising the importance of primary care as a growth area for healthcare delivery.^1^ In response, the Department of Health and Social Care identified three strategic shifts — hospital to community, sickness to prevention, and analogue to digital — to reform and transform healthcare services as part of their 10-year health plan.^2^ It is highly likely that healthcare education will have an important role in realising these priorities, thereby ensuring that the future workforce is capable of delivering high standards of care within a remodelled NHS.

The Society for Academic Primary Care (SAPC) and the Royal College of General Practitioners (RCGP) provided a comprehensive and forward-facing approach for delivering undergraduate general practice education within *Teaching General Practice* ^3^ and *Learning General Practice* in 2018 and 2021, respectively.^4^ These landmark publications highlighted some of the opportunities for delivering primary care education. Since their publication, there has been widespread adoption of different teaching approaches, especially technology-enhanced learning following the COVID-19 pandemic. However, with a major change in the configuration of the NHS on the horizon, there is further opportunity to reflect on how primary care education could be delivered in the future. In order to better align general practice education with changes in health care, it would make sense to identify innovations and interventions that fit seamlessly with these political drivers for change; and notably the three strategic shifts set out in the 10-year forward view.

## Hospital to community

The longstanding model of students spending more than three-quarters of their placement time in hospitals has been challenged.^3^ While over 90% of health care is delivered within primary care settings, around 9% of medical education is delivered in general practice.^5^ Recognising and addressing this incongruence, opportunities to expand conventional models of primary care education need to be realised. Of course, any expanded primary care curriculum would require sufficient capacity for learning, both in terms of training infrastructure and supervision.

Longitudinal Integrated Clerkships (LICs) have been gaining popularity as a mechanism for expanding learning experiences in primary care, while also providing benefits such as greater exposure to positive role modelling and team identity for learners.^6^ The concept of LICs could be evolved, and now designed using a hybrid placement approach, combining community and hospital settings throughout the primary care curriculum to provide a more authentic and holistic view of the patient journey.

*“There is now an opportunity to reconsider how primary care education curricula could be delivered within the future NHS.*”

Where there may be limited capacity within general practice, students could spend time learning in other areas of primary care, such as in care homes, pharmacies, and health hubs. Recently, there has been a growth of Community Diagnostic Centres (CDCs).^7^ CDCs are locally based and offer rich learning opportunities with a variety of diagnostics and specialties, such as radiology, gastroenterology, and pathology.^8^ These enable students to both acquire and apply procedural skills, such as point-of-care ultrasound, in a more hands-on manner. Likewise, diagnostic and data interpretation skills could be refined in-situ next to the patient, rather than from 2D images in textbooks that are decontextualised from real patients, and for the sole purpose of passing exams. Combined with other community services, such as musculoskeletal and pain management, learners can experience an integrated systems approach to health care.

## Sickness to prevention

Preventive medicine is not a new concept within primary care. The 10-year health plan, however, refocuses our attention on this and the potential opportunities to teach preventive health within the evolving healthcare system.

It may first be appropriate to re-imagine what we define as ‘prevention’. Often considered in the context of a binary choice between preventing and/or curing disease, this may now be perceived as reductionist when categorising how patients present when seeking medical attention. The definition, within medical education, could be broadened to include preventing acute admissions, re-admissions, or polypharmacy.

Similar thinking can be applied to the teaching of clinical presentations and long-term conditions. For example, heart failure is a multi-system disorder that some believe may not be best taught on a cardiology ward. Heart failure nurses deliver the majority of follow-up care for patients, making fluid balance assessments and liaising with the multiprofessional team to manage medical therapies.^9^ Following the patient through the hospital was often a phrase used to describe how best students could learn on the wards, but this concept should be re-imagined through the lens of today’s NHS and configuration of care pathways. Learning from multiprofessional teams in this way would help to ensure students better experience care and continuity within the community, and what the impact of long-term conditions have on patients in their own locality.

There is now a plethora of services in primary care that support patients when preventing ill-health and capitalising on learning experiences with newer initiatives, such as social prescribing, pharmacy-first, and community care teams enhancing preventive education. Furthermore, designing curricula with other professional groups such as Public Health would facilitate learning about core topics, such as health protection, vaccination, and screening, in a multiprofessional way that goes beyond the conventional primary and secondary care interface.

## Analogue to digital

Primary care has an established infrastructure for routinely using electronic patient records while many hospitals still rely on handwritten medical notes. There is huge opportunity to teach medical students about the effective use of digital records, and how to deploy tools embedded within general practice clinical systems, such as risk prediction, electronic prescribing, and safety alerts, to provide safe and effective care. With the advent of artificial intelligence (AI) technologies, there may be opportunity to integrate these into general practice systems to improve efficiency, productivity, and safety of care, and competently integrating AI within regulatory and ethical constraints is an important learning need for healthcare professionals.^10^

Opportunities now exist for using more innovative technologies to deliver primary care education, and over the last 5 years there has been a rapid increase in deployment of technology-enhanced learning (TEL) in undergraduate medical curricula.^11^ There is a growing evidence base for using live-streaming, augmented, and virtual-reality technologies, for teaching clinical reasoning and communication, while also providing exposure to authentic clinical experiences.^12^ Despite technologies often being deployed outside of clinical environments, TEL affords the possibility of developing more complex competencies, having evolved from use in postgraduate education for clinical skills. While TEL comes with its own challenges, it does provide opportunity to increase the consistency and scalability of learning without space and capacity boundaries.^12^ Consequently, there is now a need to ensure that general practices and other community-based settings are digitally ready to deliver effective training to learners and health care to patients in order to meet future population needs.

The NHS is constantly evolving and becoming increasingly complex. There is now an opportunity to reconsider how primary care education curricula could be delivered within the future NHS. Utilising political drivers and priority areas for change may help open our thinking into opportunities for teaching and learning that may previously have been overlooked or embryonic in their development. In doing so, medical educators may be able to better bridge the dichotomy between delivering higher education within a complex NHS, while also ensuring that education is rooted in the future vision of healthcare services.
